# From a Coriander Mayonnaise to a Vegan Analogue: Assessing pH and Salt Influence in a *Saccharomyces cerevisiae* Yeast Protein Extract and *Chlorella vulgaris* Mixed System

**DOI:** 10.3390/foods14040587

**Published:** 2025-02-10

**Authors:** Pedro Coelho, Carmo Serrano, Norton Komora, Anabela Raymundo

**Affiliations:** 1 Linking Landscape, Environment, Agriculture and Food (LEAF), School of Agronomy, Lisbon University (ISA), Tapada da Ajuda, 1349-017 Lisboa, Portugal; pmcoelho@isa.ulisboa.pt (P.C.);; 2 Mendes Gonçalves SA, Zona Industrial, Lote 6, 2154-909 Golegã, Portugal

**Keywords:** mayonnaise, yeast protein extract (YPE), microalgae *C. vulgaris*, pH, salt

## Abstract

History aside, traditional mayonnaise faces a rising animal welfare concern dietary approach and remains dependent on cold environments throughout the supply chain due to food safety. Nowadays, consumers are able to find alternative formulas from vegetable sources with relevant emulsifying capacity. However, sensory characteristics may differ from the traditional expected product. A mixed system composed of the innovative ingredient heterotrophic white *Chlorella vulgaris* and a disruptive emulsifier, yeast protein extract (YPE), was assessed to transform traditional coriander mayonnaise into an analog product. The effect of pH and salt (NaCl) content was also evaluated. The mixed system depicts a promising stability since the average Sauter diameter of both is similar (7.94 μm and 7.49 μm), also observed in the unimodal droplet size distribution. Viscoelastic behavior has slightly different responses for the plateau model (278.951 Pa and 252.053 Pa), while increasing the salt content reflects an approximation regarding firmness (0.059 N and 0.057 N) and adhesiveness (0.372 N.s and 0.361 N.s). Introduction of microalgae increases bioactivity, mainly TPC (+118.84 ugGAeq/g) and antioxidant activity—RSA (+31.29 ugTEAC/g) and FRAP (+35.26 ugTEAC/g). Despite the color deviation, the sensorial analysis of both products enlightened the absence of major perception.

## 1. Introduction

The food sector raises a contemporary concern driven by sustainability issues, mainly combining nutritious food offers and limited natural resource management. Growing trends align with the preference and consumption of foods containing plant-based proteins [1]. This shift generates opportunities for food researchers and companies to lead disruptive innovation. Flexitarian lifestyle consumers may lead the promotion of nonconventional alternative foods consumption if the circle of research-industry-consumers respect a clear pact of transparent and helpful information associated with a competitive price [2]. New millennium research approaches mayonnaise production in order to develop similar products from alternative nonanimal proteins, facing a rising need to find valid options for egg proteins [3]. Current challenges remain around sensory characteristics as aroma, color, flavor, and texture. Alternative protein incorporation may very well improve the physicochemical, nutritional, and functional properties of mayonnaises [4]. Recent studies have found potential in plant-origin protein mixed systems incorporated in emulsions, such as grass pea sweet miso with lupin and faba bean [5] and white fruit flour from apple, nectarine, pear, and peach with lupin and faba bean [6]. Another study compared emulsions produced with solo proteins of aquafaba, faba bean, green pea, and lupin [7]. Other disruptive approaches studied analogues of proteins like single-cell proteins from the yeast *Saccharomyces cerevisiae* [8], the green-colored cyanobacteria *Spirulina* [9], and the insect *Tenebrio molitor* [10]. These range of ingredients depict potential as emulsifiers based on their high protein content, alongside the possible nutritional boost in terms of bioactive compounds and minerals.

In traditional mayonnaise, egg fractions have an important emulsifier role. Egg yolk is a standard ingredient, mainly due to the presence of lecithin, which is a complex mixture of phospholipids acting as a natural emulsifier [11]. Egg white is also commonly added because of its rich nutritional value and functional properties, specifically emulsifying, foaming, gelling, and thickening [12]. Whole or single fraction addition can be in liquid or powder form. An emerging eco-friendly consciousness among the new generation of consumers raises questions towards the food industry about food safety and security, sustainability, and animal welfare. Representing a historical and widely consumed sauce, traditional mayonnaise composition complies with these emerging concerns. At first, the addition of unpasteurized eggs concerned consumers’ health in terms of food safety and quality, before a gap of unmonitored contamination risk by *Salmonella* [4]. Secondly, this emulsion-based sauce’s components are strictly dependent on refrigerated supply chains, requiring impeccable quality control parameters to avoid microbial contamination [13]. Thirdly, containing a high proportion of unsaturated fats, mayonnaise becomes susceptible to oxidation acceleration in the presence of small quantities of transition metals, further developing off-flavors [14]. A mitigation strategy resides in the addition of antioxidants such as rosemary extract and Ethylenediaminetetraacetic acid (EDTA). Although these are not naturally present in mayonnaise components, they work as a chelating agent that sequesters ions vulnerable to oxidation [15].

The present work focuses on the substitution of animal protein in readily available coriander mayonnaise with a mixed system of *Saccharomyces cerevisiae* yeast protein extract (YPE) and white *Chlorella vulgaris* (Microalgae). In one hand, YPE stands recently as an emerging novelty in both food and feed, representing a disruptive source of both macro and micronutrients, a versatile flavoring agent controlled by Maillard reactions, and a production line processes enhancer such as reducing yogurt fermentation time, fining wines, and extending processed meat products physical properties [16,17]. Regarding its many applications and technological advantages, yeast proteins are considered safe and economically accessible for human consumption [18]. Preliminary studies state that *S. cerevisiae* contains polymers capable of emulsifying and stabilizing emulsions [19]. Yeast extract emulsions’ kinetic stability is guaranteed by the droplets’ electrostatic repulsion, viscoelastic behavior, and resemblance to pickering-style stabilization. These mechanisms increase the emulsion resistance against physical instabilization phenomena [20]. On the other hand, microalga are not only suitable for inclusion in daily human diets to enhance nutritive intake [21], but they also contain functional properties capable of stabilizing oil-in-water emulsions [22]. Microalga’s natural composition favors new sensory experiences, offers functional properties with technological interest, prevents oxidation and color change processes in foods, and aligns with sustainable production and harvesting sources. Several studies underline the potential of *Chlorella vulgaris* as a dietary supplement with a positive impact on human health maintenance. Nutritive and vitaminic richness in *C. vulgaris* composition relates to antidiabetic, antihyperlipidemic, antihypertensive, antioxidant, and immunomodulatory properties [23]. Chlorella *vulgaris* is a heterotrophic microalga, requiring certain levels of sunlight exposure in a CO_2_ environment to attain necessary sources of energy and carbon [24].

Therefore, microalga can be considered a healthy substitute for the lipoproteins in egg yolk and can also work as a natural substitute for antioxidants and colorants, making it possible to obtain greener Clean Label products. The recent decade rose among the consumers a food philosophy guided by healthy eating habits, motivating the consumption of safe foods with a valid nutritive value to support the immune system [25]. Developed countries’ societies lean towards Clean Label products, under the values of convenience, health concerns, and sustainability. Convenience refers to a rising consumption of precooked or ready-to-eat meals. Health concerns focus on fighting unbalanced consumption habits related to diseases and allergies/intolerances towards certain types of food components. Sustainability corroborates with a greater acceptance of the climatic changes due to constant pollution from conventional agricultural and nonorganic food production practices [26]. Clean Label products are responsible for reducing the number of ingredients in their formulation, substituting most of their synthetic components with natural sources, and sharing transparency with the consumer. As far as consumers’ perception is concerned, coloring agents and stabilizer agents are less natural ingredients [27]. A mixed system of YPE and microalgae biomass (heterotrophic white *Chlorella vulgaris*) may change the perception of the consumer and answer the contemporary gap between consumption intent and market availability. This emulsifying system was evaluated, as well as the impact of pH and salt on the stability of the emulsified system.

The main goals of the following research are the development of a vegan clean label coriander mayonnaise analog with overall improvements associated with the mixed system application and the study of possible physical changes in the final product due to unforeseen pH and salt concentration deviations during the production chain.

## 2. Materials and Methods

### 2.1. Materials

Main ingredients were supplied by Casa Mendes Gonçalves (Golegã, Portugal), in order to mimic their coriander mayonnaise formula: colza oil, alcohol vinegar, modified starch, egg yolk powder, salt, sugar, coriander, colorant (shade yellow; E-104), potassium sorbate (E-200), citric acid (E-330), xanthan gum (E-415) and ethylenediaminetetraacetic acid (EDTA; E-385). Usually, the company produces the sauces with sunflower oil; however, due to the recent Russia–Ukraine war, global markets were affected, and the price of vegetable oils rose [28], directly impacting the food industry. Yeast protein extract (M2344-1) retrieved from *Saccharomyces cerevisiae* was kindly supplied by Proenol SA (Canelas, Portugal), a biotech Portuguese company leading the supply of this ingredient to continuous research focused on its application in food products. Heterotrophic white *Chlorella vulgaris* results from controlled environment mutagenesis [29], produced by Allmicroalgae (Pataias, Portugal).

### 2.2. Methods

#### 2.2.1. Mayonnaise Production

**Figure A1:**
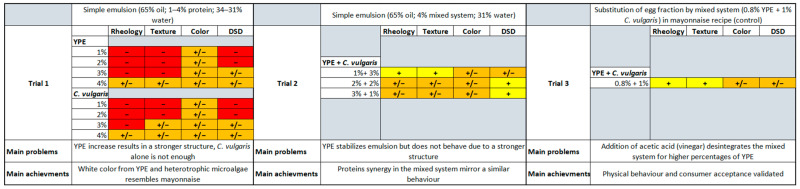
Initial attempts to assess YPE and microalgae concentration to produce food emulsions under industry predetermined requirements.

The control formula’s main components were disclosed by the company Casa Mendes Gonçalves in order to study alternative sustainable proteins to produce an analog to the market-available coriander mayonnaise sauce. To achieve a free animal food product, egg yolk powder was substituted by a mixed system (CvY) composed of 0.8% of *Saccharomyces cerevisiae* yeast protein extract and 1% heterotrophic white *Chlorella vulgaris*. After a battery of attempts with different percentages in mixed systems for each protein (Figure A1), the present analysis focused on a concentration that better fitted the requirements from the company in comparison with the current target (Control). Also, changes in pH and salt concentration were assessed to study the full extent of its influence in terms of rheological response. Batches were produced in a Thermomix kitchen robot (Vorwerk, Wuppertal, Germany), under the following settings: temperature = 20 °C, medium velocity = 4 position, and emulsification time = 40 s. The final formula in Table 1 was applied in 1 kg batches for an initial scale-up viability assessment.

Powdered sources of proteins, white *C*. *vulgaris* biomass and *S*. *cerevisiae* (YPE), were hydrated [8] and homogenized in distilled water upon a AREX heating magnetic stirrer (VELP Scientifica Srl, Usmate, Italy) for 30 min at room temperature. Before emulsification, the remaining dried ingredients were added to the previously hydrated mixed system. Emulsification occurred with the colza oil pouring, followed by vinegar addition. Each mayonnaise was analyzed on the following day in terms of pH, rheology behavior, texture, color, and droplet size distribution. Antioxidant potential and total phenolic compounds quantification were also assessed.

#### 2.2.2. Physicochemical Characterization

Rheological characterization was carried out using a rotational rheometer, the Haake Mars III Modular Advanced Rheometer System (Thermo Fisher Scientific Inc., Karlsruhe, Germany), connected with the Peltier cooling system (Thermo Fisher Scientific Inc., Karlsruhe, Germany) and the air compression system Eheim professional (Eheim, Deizisau, Germany).

Frequency sweep tests were performed with an ensemble of a conic probe and plate system. Steady-state flow measurements were performed with a serrated parallel plate system with a 1.5 mm gap. Both ensemble systems had 35 mm, and tests ran at 20 °C.

##### Viscoelasticity

Frequency sweep tests, or mechanical spectra, were assessed by means of small amplitude oscillatory shear (SAOS) measurements within the predefined linear viscoelastic region—LVR (accessed by a stress test at 1 Hz) for a frequency range of 0.01–100 Hz. The output refers to the mechanical spectrum (G’ and G” as a function of frequency) and the loss tangent (tan δ = G”/G’). The plateau modulus (G^0^_N_) was also estimated as the value of G’ observed for the minimum value of the loss tangent [30].

##### Viscosity

Viscosity tests were assessed by means of steady-state flow curves in a shear rate range of 10^−4^ to 500 s^−1^. The Williamson model (1) was applied to adjust the test outputs using the Origin 2019 software (OriginLab, Northampton, MA, USA) for a better understanding of the viscosity *versus* shear rate curves [31].

(1) $$\eta =\frac{\eta_{0}}{1+{\left(k\dot{\gamma }\right)}^{m}}$$

In the Williamson model (1), η_0_ is the zero shear rate limiting viscosity at low shear rates (Pa.s); *k* is the consistency coefficient (s) and a dimensionless shear-thinning index; and m is the slope of the power law shear-thinning region. Both tests were carried out in triplicate.

##### Texture Evaluation

To compare the final results, a texture profile analysis (TPA) was performed in a TA.XT plus texturometer (Stable Micro-Systems, Godalming, UK). TPA was performed with a 15 mm double penetration at 1 mm/s of a 19 mm diameter cylindrical acrylic probe in cylindrical glass jars (100 mL with 66 mm diameter, 56 mm height) filled with each sample up to 32 mm in height. Each sample was tested 5 times at room temperature. The main test outputs are firmness (N) and adhesiveness (N.s).

##### Evaluation of the Droplet Size Distribution

Droplet size distribution (DSD) was assessed to achieve an initial correlation with the final product structure and physical stability. Tests were carried through a Partica LA-960V2 laser scattering particle size distribution analyzer (Horiba, Kyoto, Japan). The minimal necessary portion of emulsion was introduced as per the equipment software’s running test indication. Test outputs are the *d*_3,2_ (2) and *d*_4,3_ (3) diameters, also span (4), after a triplicate measurement.

Sauter diameter (*d*_3,2_):

(2) $$d_{3,2}=\frac{\sum n_{i}d_{i}^{3}}{\sum n_{i}d_{i}^{2}}$$

De Brouckere diameter (*d*_4,3_):

(3) $$d_{4,3}=\frac{\sum n_{i}d_{i}^{4}}{\sum n_{i}d_{i}^{3}}$$

*Span*:

(4) $$Span=\frac{d\left(90\right)-d(10)}{d(50)}$$

In terms of diameters, *n_i_* represents the number of droplets with a diameter equivalent to d_i_ [5]. While *d*_3,2_ indicates the overall droplets’ average diameter, *d*_4,3_ indicates particle size changes caused by an increased sensibility to destabilize processes, such as droplet aggregation [32]. In terms of span, *d*(x) is the x volume percentile of droplets with diameters smaller or equal to the predefined values, meaning that a higher value of span indicates a diverse droplet polydispersity [33].

##### Color Comparison and pH Measurement

Color differences and comparisons were assessed based on the CIELAB color coordinate system (L*, a*, b*). Coordinate parameter (luminosity and chromatic range) measurements were analyzed through a Chroma Meter CR-400 colorimeter (Konica Minolta Business Technologies, Inc., Tokyo, Japan) calibrated according to the Konica Minolta white standard: Y = 86.7; x = 0.3160; y = 0.3233. The total color change (∆*E*) was calculated between microalgae samples and the target using the following Formula (5):

(5) $$∆E=\sqrt{{\left(L_{i}^{*}-L_{0}^{*}\right)}^{2}+{\left(a_{i}^{*}-a_{0}^{*}\right)}^{2}+{\left(b_{i}^{*}-b_{0}^{*}\right)}^{2}}$$

For each sample, 10 replicates were considered at different points of the sample spread in a petri dish.

To determine the final pH, samples were measured in triplicate with a pH electrode connected to a phM92 Lab Meter (Radiometer, Copenhagen, Denmark). Equipment calibration was performed with standard buffer solutions at pH 4, 7, and 9.

#### 2.2.3. Antioxidant Activity

##### Extraction Methodology

Antioxidant activity was assessed through 1,1-diphenyl-2-picrylhydrazyl (DPPH) Radical Scavenging Activity (RSA) and Ferric Reducing Antioxidant Power (FRAP) methods. Total Phenolic Compounds (TPC) were measured by the Folin–Ciocaltêu (FC) methodology. Sample preparation began with an extraction previously adapted and optimized to assess microalga food products antioxidant activity [34]. To achieve both aqueous and oil phase, 2 g of sample + 2 mL of diluted Methanol (Honeywell, Morris Plains, NJ, USA) in deionized water (MeOH:H_2_O/70:30 ratio) + 2 mL of Hexane (Honeywell, Morris Plains, NJ, USA) were mixed for 1 h in a Reax 2 shaker (Heidolph Instruments, Schwabach, Germany) and duly centrifuged in a Z383-K high performance centrifuge (Labnet International, Edison, NJ, USA) at 5000 rpm/4 °C/10 min. After centrifugation, a hydroalcoholic fraction (supernate) was recovered, and the process was repeated in triplicate. The recovered fraction was evaporated in volumetric glass flasks submerged in a 40 °C bath through a R-300 Rotavapor^®^ (BUCHI, Barcelona, Spain) connected to a V-700 vacuum pump (BUCHI, Barcelona, Spain) reaching 58 bars. The evaporation process resulted in dry matter for each fraction. Each dry matter was weighted and dissolved in 2 mL of Dimethyl sulfoxide (DMSO) (Fisher Scientific International, Inc., Pittsburgh, PA, USA) in an LBX V05 vortex stirrer (Labbox Labware, Barcelona, Spain) and further filtered through a syringe coupled to a 0.20 μm nylon filter with a 25 mm diameter.

##### DPPH

The Radical Scavenging Activity assay was performed as previously mentioned [35] and adjusted for microalga food products research [34]. A combination of 20 μL of the previously extracted sample with 180 μL of a methanolic DPPH solution in a concentration of 60 μM was placed in NUNCTM 96-well microplates (Nalge Nunc International, Rochester, NY, USA) and underwent an incubation period for 30 min. Afterward, absorbance was measured at 517 nm in a Libra S22 UV/Visible Spectrometer (Biochrom, Cambridge, UK). Analysis outputs were expressed as Trolox equivalents per gram (μgTroloxEq/g).

##### FRAP

The Ferric Reducing Antioxidant Power assay consists of the reduction of Fe^3+^-TPTZ (ferric-tripyridyl triazine complex) to Fe^2+^ (ferrous complex), which is noticeable due to a color change under an intense blue spectrum [36]. FRAP solution was prepared with 10 mL of acetate buffer (300 mM), pH adjusted to 3.6 using acetic acid, blended with 1 mL of ferric chloride hexahydrate (20 mM) dissolved in distilled water, and 1 mL of 2,4,6-Tris(2-pyridyl)-s-triazine (TPTZ—10 mM) dissolved in chloridric acid (HCl—40 mM). The analysis was adapted as previously described [37] with minor changes to enable execution in 96-well microplates. Inserted in the NUNCTM 96-well microplates (Nalge Nunc International, Rochester, NY, USA), 25 μL of the previously extracted sample combined with 175 μL of the FRAP solution (prewarmed to 37 °C) was left in triplicate to incubate for 30 min away from light at room temperature. Afterward, absorbance was measured at 595 nm in a Libra S22 UV/Visible Spectrometer (Biochrom, Cambridge, UK). Analysis outputs were expressed as Trolox equivalents per gram (μgTroloxEq/g).

##### Total Phenolic Compounds (TPC)

The Total Phenolic Compounds (TPC) assay followed the 96-well microplate Folin–Ciocalteu (FC) (PanReac AppliChem, Barcelona, Spain) technique [38], previously adjusted and optimized to microalga extracts [34]. Test preparation started with the addition of 100 μL of FC reagent (1:4) to 20 μL of the extract and a settle time of 5 min at room temperature. Each triplicate was added 80 μL of 7.5% sodium carbonate solution, and the microplate was kept for 2 h in the dark at room temperature. The absorbance was measured at 760 nm using a Thermo Scientific™ Multiskan™ GO Microplate Spectrophotometer (Fisher Scientific International, Inc., Pittsburgh, PA, USA). Analysis outputs were expressed as gallic acid equivalents per gram (μgGAEeq/g).

#### 2.2.4. Sensorial Analysis

Acceptability and purchase intent towards the final product have a major influence on scale-up viability. For this purpose, a ranking test based on ISO 8587:2006 was firstly performed with 20 voluntary participants [39], qualitatively targeting the main organoleptic parameters (aspect, color, flavor, odor, texture, global evaluation) in a hedonic scale between 1 (extremely dislike) to 9 (extremely like). Secondly, the purchasing intent for each product was asked in a quantitative range from 1 (Certainly would not buy) to 5 (Certainly would buy). Both egg mayonnaise (Control—378) and mixed system microalgae + YPE mixed system mayonnaise (CvY—259) were taken into comparison with random codes. The tasting panel was run by the common standards used within the research center LEAF (Linking Landscape, Environment, Agriculture and Food) from Instituto Superior de Agronomia, Portugal. Volunteer participants were provided with an informed consent in accordance with the ethical standards of the local committee responsible for human experimentation and with The Code of Ethics of the World Medical Association (Declaration of Helsinki of 1975, as revised in 2013).

#### 2.2.5. Statistical Analysis

Results obtained in the conducted analysis were submitted to statistical treatment in order to ascertain the significant differences between the output averages. Statistical analysis was performed in GraphPad Prism software (version 9.0) to carry out the analysis of variance (ANOVA), using Fisher’s test to compare two samples and the Tukey’s test to compare three or more samples. Both tests used a 95% confidence level (α = 0.05). Different letters indicate significantly different results (*p* < 0.05) in the data presented.

## 3. Results and Discussion

### 3.1. pH Measurement

The pH value of each sample was measured to achieve a clearer comprehension of its impact in the following analysis. The stable pH for each sample is shown in Table 2.

The pH of the Control does not present significant differences compared to the microalgae + YPE systems with the usual citric acid concentration (0.22 g/100 g) or when NaCl is added at concentrations of 0.1 or 0.5 g/100 g. However, in cases where citric acid addition was increased or decreased, there is a significant alteration in the stable pH values of the emulsions, proportional to the concentration of acid added (0.1 or 0.4%).

### 3.2. Rheological Behaviour

#### 3.2.1. Frequency Sweeps

**Figure 1:**
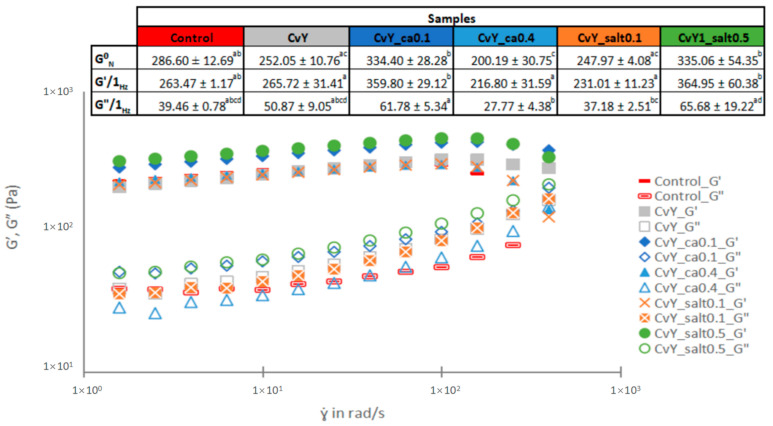
Mechanical spectra and plateau model (G^0^_N_), storage (G’), and loss (G”) moduli at 1 Hz (6.28 rad/s) comparison between Control and samples with the mixed system (CvY). Different letters indicate significantly different results (*p* < 0.05) for each row according to Tukey’s test.

From the mechanical spectra (variation of G’ and G” in the LVR). The storage modulus corresponds to the elastic capacity associated with a solid-like behavior of viscoelastic fluids, whereas the loss modulus corresponds to the thick capacity associated with a liquid-like behavior [40]. Overall, G’ values present higher values than the G’’ values, indicating a common elastic behavior. Both moduli depict a gradual increase along the frequency, as observed in Figure 1, leading to an expectation of a stable emulsion with a gel-like behavior [7].

By substituting egg yolk (Control) with microalgae + YPE (CvY), it is possible to achieve a similar viscoelastic behavior (similar shape of the spectra). However, the YPE promotes an increase in the complexity of the entanglement network established between the protein films of neighboring droplets, enhancing their structural interactions and overall system stability, which is reflected in higher plateau modulus (G^0^_N_) values [6].

The control emulsion and the mixed systems CvY, CvY_ca0.4, and CvY_salt0.1 exhibit G’ and G’’ (1 Hz) values, as well as G^0^_N_ values, without significant differences (*p* < 0.05). These results suggest that the physical stability of these emulsions is similar, as evidenced by their comparable viscoelastic properties. Both viscoelastic functions are critical parameters in predicting the stability and performance of emulsions under stress [41]. The plateau modulus (G^0^_N_) further supports the network structure’s resilience, a key factor in determining the long-term stability of such systems. These findings align with the established principles of soft matter rheology, particularly in systems where the balance between elastic and viscous components plays a pivotal role in maintaining structural integrity under various conditions [42].

It is important to highlight that the mixed systems exhibit a synergistic effect [43], making it possible to obtain emulsions that are structurally similar to the control emulsion prepared with egg. This highlights the potential of these systems as an important approach for the development of vegan emulsions, aligning with the growing consumer trend toward plant-based and sustainable food products. The increasing demand for vegan alternatives reflects a shift in dietary preferences driven by environmental concerns, health benefits, and ethical considerations. Similar studies have demonstrated the successful use of other plant-based proteins in vegan emulsions, such as soy protein [44], pea protein [45], lentil protein [46], and lupinus and fava proteins [7], showcasing their ability to stabilize emulsions while offering functional and nutritional benefits.

Although, from an industrial perspective, the pH values and salt concentration are typically well-defined according to the specifications of a given product, it is crucial to understand the sensitivity of emulsified systems to variations in these two parameters. Such knowledge is fundamental for optimizing formulation stability, especially under processing and storage conditions where fluctuations in pH or ionic strength can impact the emulsions’ rheological properties, microstructure, and shelf life [47,48]. Insights into these sensitivities contribute to the development of more robust and versatile formulations suitable for a wide range of applications.

With respect to pH variation, two conditions were studied: CvY_ca0.1 (pH—4.10 ± 0.03) and CvY_ca0.4 (pH—3.65 ± 0.02), with citric acid concentrations of 0.1 and 0.4 g/100 g, respectively. The pH values obtained were significantly different, and the system with the lower pH exhibited a significant reduction (*p* < 0.05) in the viscoelastic properties compared to the other conditions studied. This behavior may be attributed to the pH approaching the isoelectric point of the mixed system, where interactions between protein particles become less favorable due to charge neutralization. Similar behavior has been observed by other authors; for instance, in soy protein emulsions, lower pH values resulted in decreased viscoelastic properties [49]. Likewise, Vélez-Erazo et al. [50] reported that a reduction in pH in pea protein emulsions led to a significant decrease in gelation and stability properties, which was associated with the proximity to the isoelectric point. This behavior reflects changes in protein interactions, which directly affect the formation and stability of emulsions. In addition, emulsions’ rheological response may also be adjusted by the pH, probably due to changes in the proteins’ electrostatic interactions [51].

To evaluate the impact of salt (NaCl) addition on the rheological behavior of emulsions, two concentrations were studied: 0.1 (CaY_salt0.1) and 0.5 (CaY_salt0.5) g/100 g. It was observed that the emulsion with higher salt content showed an enhancement in its structure, corresponding to higher values of G’, G”, and G^0^_N_. This indicates a strengthening of the emulsion network, likely due to the increased ionic strength. Salt content’s main impact in both moduli evidences a reinforcement in the viscoelasticity capacities, ultimately improving final structure and stability [52].

The presence of salt influences the electrostatic interactions between protein molecules, which can lead to changes in the protein–protein network, enhancing the stability and rheological properties of the emulsions. Similar results have been reported in other studies; for example, Sriprablom et al. [40] found that increasing the ionic strength in whey protein-stabilized emulsions enhanced their viscoelastic properties, attributed to the screening of electrostatic repulsions between protein molecules. Additionally, Mańko-Jurkowska and Domian [53] observed that higher salt concentrations in chickpea protein emulsions resulted in stronger gel formation and improved stability due to the ionic crosslinking effect, which promoted better structuring of the protein network. Such findings highlight the critical role of ionic strength in emulsion formation and stabilization.

#### 3.2.2. Viscosity Measurements

**Figure 2:**
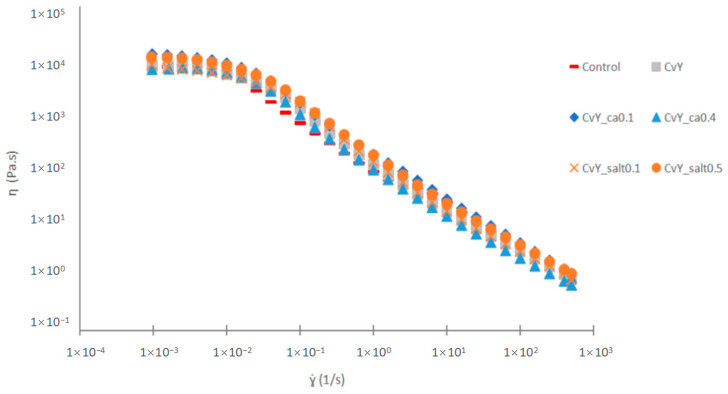
Flow curves and Williamson model adjustment parameters comparison between samples: Control; CvY; CvY_ca0.1; CvY_ca0.4; CvY_salt0.1; and CvY_salt0.5.

Both the control and mixed systems of CvY mayonnaises’ steady-state flow curves depict a shear-thinning behavior, with an expected tendency towards a zero-shear rate-limiting viscosity. This behavior is closely aligned with the oil droplet deflocculation mechanism [54]. In Figure 2, it is possible to compare the behavior of the Control and CvY, as the tests were performed with different concentrations of citric acid and salt (NaCl).

Adjusted Williamson curves parameters are presented in Table 3.

The control and the emulsion with the binary system YPE + microalgae (CvY) do not show significant differences (*p* < 0.05) in the values of zero-shear rate limiting viscosity (η_0_). This suggests that both systems exhibit similar flow behavior under conditions of very low shear rates, where the material is dominated by its intrinsic viscosity and does not undergo shear thinning. Such behavior is often observed in complex systems, where viscosity at low shear rates is an important indicator of the network structure and molecular interactions [55].

When testing the impact of pH on the viscosity (CvY_ca0.1 and CvY_ca0.4), it was found that the results obtained are consistent with those observed in terms of linear viscoelastic behavior. That is, the more acidic sample—CvY_ca0.4 with a pH value of 3.65 ± 0.02—shows a significantly (*p* < 0.05) lower value of η_0_. This result aligns with the previous observation; the pH value reached is close to the isoelectric point of the mixed system. In such situations, the limiting viscosity of emulsions should be lower, as the protein–protein interactions are weakened due to charge neutralization at the isoelectric point, resulting in a less structured network [56]. These changes in viscosity can be attributed to the reduced ability of proteins to form stable networks at pH values close to their isoelectric points.

Regarding salt concentration, no significant differences (*p* > 0.05) were observed between the two NaCl concentrations studied (0.1 and 0.5 g/100 g). This result does not align with what was observed in terms of the mechanical spectra, where a higher NaCl concentration induced an increase in the degree of structuring of the emulsion. However, this increased structuring did not impact the flow behavior. A similar situation was observed by other authors. Quintana et al. [57] reported that emulsions stabilized by whey protein isolate exhibited enhanced viscoelastic properties at higher ionic strengths, but the zero-shear viscosity remained unchanged. Similarly, Sriprablom et al. [40] demonstrated that whey protein stabilized emulsions showed increased gel-like behavior under higher salt concentrations, although the flow properties, such as shear-thinning behavior, remained consistent across different salt levels. These findings suggest that while ionic strength can significantly influence the network structure, it may not always translate to changes in bulk flow behavior.

### 3.3. Texture Profile Analysis (TPA)

Texture parameters obtained from the TPA profile are relevant aspects that increase the probability of alternative products’ success among consumers. This relies on the perceived texture during the tasting experience. Perceived texture splits into three moments. The first moment refers to the extra oral, mainly dependent on appearance. The second moment refers to the intra oral, characterized by first mandibular compression, chewing action, and the swallowing process. The third moment relates to the physical behavior of the product during ingestion, mainly in parameters as hardness, fracturability, viscosity, and adhesiveness [27]. TPA parameters were compared based on Table 4.

With the exception of the CvY_ca0.1, all the mixed systems depicted a significant decrease in firmness and adhesiveness when compared with the Control. Adding microalga, especially *C*. *vulgaris*, seems to generate softer textures in the food products [58]. This behavior does not reflect what was observed in terms of linear viscoelastic behavior, as in this case, the emulsions CvY_ca0.1 and CvY_salt0.5 exhibited a higher degree of structuring compared to the control, which should correspond to higher firmness values. However, firmness results do not always reflect the behavior evaluated under oscillatory conditions for a low range of applied stresses. This discrepancy arises because the types of stresses applied are fundamentally different. Similar discrepancies have been reported by other authors. Chen and Dickinson [59] observed that emulsions with enhanced viscoelastic moduli in oscillatory tests did not exhibit corresponding increases in firmness during large deformation tests due to differences in the nature of applied stress. Additionally, protein-stabilized emulsions exhibited stronger network structures under oscillatory shear, but this structural enhancement did not translate to firmness measurements under large deformation conditions, highlighting the importance of the testing methodology.

The elasticity values of the emulsions do not deviate from the Control. This texture parameter is not appropriate for discriminating the behavior of emulsions, as noted by other authors. For example, Graça et al. [60] demonstrated that firmness and adhesiveness were sufficient for characterizing emulsions, emphasizing that these textural parameters provide a more direct assessment of the structural properties and stability of such systems.

Cohesiveness is also not usually a relevant parameter for discriminating emulsions. It is observed that there is a general decrease of cohesiveness in the mixed systems, compared with the control.

### 3.4. Droplet Size Distribution (DSD)

Assessing the DSD of the emulsions requires an intrinsic comparison of both the Sauter diameter (d_3,2_) and the De Brouckere diameter (d_4,3_). The Sauter diameter, or surface-area-weighted mean diameter, provides insights into the ratio of the total droplet volume to the total surface area, which is critical for understanding the efficiency of emulsifier coverage. In contrast, the De Brouckere diameter, or volume-weighted mean diameter, reflects the contribution of larger droplets to the overall size distribution, which significantly affects stability and coalescence behavior. The combined analysis of these two parameters allows for a comprehensive understanding of emulsion characteristics and has been widely used in emulsion science [61].

**Figure 3:**
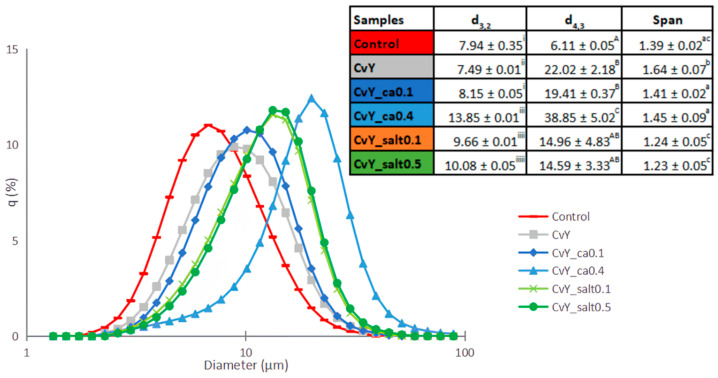
Droplet size distribution, Sauter diameter (d_3,2_), De Brouckere diameter (d_4,3_), and span comparison between samples: Control; CvY; CvY_ca0.1; CvY_ca0.4; CvY_salt0.1 and CvY_salt0.5. Different letters indicate significantly different results (*p* < 0.05) for each column according to Tukey’s test.

From the d_3,2_ measurement, it is possible to obtain the average diameter for most droplets, where smaller values mean smaller and condensed droplets, mirroring a more stable system [62]. From the d_4,3_, or volumetric diameter, it is possible to evaluate the susceptibility to droplet size changes linked to destabilizing processes or emulsion stability physical degradation [32]. The droplet size distribution of all samples was analyzed (Figure 3), and from the curves, the d_3,2_ and d_4,3_ values were calculated.

It was observed that all emulsions prepared with the mixed system CvY exhibited a unimodal distribution, similar to the control. This type of distribution is generally associated with more stable emulsions. A unimodal distribution indicates a more uniform droplet size, which minimizes differences in droplet behavior under stress conditions and reduces the likelihood of phenomena such as creaming, coalescence, or Ostwald ripening. Uniform droplet sizes also promote efficient emulsifier coverage, enhancing the kinetic stability of the emulsion. This relationship has been reported by McClements [63], who noted that emulsions with a narrower and unimodal droplet size distribution tend to exhibit better physical stability due to the uniformity in their interfacial properties.

On the other hand, it is observed that the smaller the average droplet diameter, the greater the stability of the emulsion. This is because smaller droplets have a higher surface-to-volume ratio, which enhances the effectiveness of emulsifier molecules in stabilizing the interface and reduces the driving force for coalescence. Additionally, smaller droplets are less prone to creaming due to their slower rise velocity, as described by Stokes’s law. Xue et al. [64] demonstrated that emulsions with smaller droplet sizes exhibit prolonged stability, attributed to their ability to resist destabilization mechanisms like coalescence and Ostwald ripening more effectively.

The auter diameter of the CvY is slightly smaller than the Control. CvY_ca0.1 is smaller than CvY_ca0.4. This decrease may be linked with the slight increase in viscosity. Higher viscosity values correlate with a lower probability of coalescence [65].

Changes in the droplet size between emulsions with different pH levels reflect a decrease in the Sauter diameter (d_3,2_) as pH increases. Decreasing the pH results in larger Sauter diameters, a fact closely related to the protein’s lower diffusion rate around the O/W interface, explained by a raise in the denaturation degree of the protein. In contrast, increasing the pH leads to lower droplet sizes [54].

Moreover, decreasing the pH reduces the electrostatic repulsion, possibly influencing the molecular conformation functional properties since the molecules bind to negatively charged groups, further decreasing the number of similarly charged groups. This interchange explains the narrow distribution observed [66]. Based on this, stronger emulsion stability can be expected with higher values of pH when the droplet size is smaller. The good stability of the emulsions at higher pH values can be attributed to their strong electrostatic repulsion [67].

Different salt concentrations deviate from the Control since it is possible to observe larger droplets. This tendency may be explained by the interaction of salt and the particle-interface mixed system attraction during mixing, resulting in a lack of particle capacity to adsorb to the interface when compared with the presence of egg yolk or due to the particle trapping energy decrease [68]. Despite the differences between the Control and CvY with different salt concentrations, these concentrations did not show different values, suggesting that salt does not have major effects on the droplet size distribution [57,69]; however, they may differ in terms of emulsion stability. Nonetheless, salt does not influence the ionic strength on the physicochemical stability of O/W emulsion, meaning that the main dependency remains in the ionic species and ionic strength of the continuous phase [70].

### 3.5. Antioxidants and Bioactive Compounds

Mayonnaise lipid portion is susceptible to oxidative deterioration derived from the auto-oxidation of unsaturated and polyunsaturated fats oil fraction. This oxidation extent may result in negative organoleptic impacts on flavor, aroma, color, and nutritional value [71]. The food industry plays a crucial role in the application of effective strategies against the lipid oxidation of mayonnaise, mainly through the addition of antioxidants or the use of other lipid sources that are naturally rich in compounds capable of antioxidant activity [72,73]. Commercial mayonnaise usually resorts to EDTA as an antioxidant and preservative component [74]. Antioxidant activity potential was compared in Table 5. While the Control contains EDTA, the mixed system CvY relies on the properties of the heterotrophic microalgae white *C. vulgaris*’s natural antioxidants.

It is possible to underline the enhancement of bioactive compounds capable of antioxidant activity in mayonnaises obtained from the mixed system YPE + *C*. *vulgaris*. The most noticeable enrichment belongs to phenolic compounds, favorable to inhibit hydro-peroxides formation [75]. These bioactive compounds are positive not only for their antioxidant capacities but also for their antihypertensive potential. However, from the nutritional perspective, microalga in general still lack proper evidence of bioactive compounds’ bioavailability after ingestion in the gastrointestinal tract [76].

A pertinent factor is connected with the viability of achieving greater antioxidant power if the amount of *C*. *vulgaris* incorporation increases [77]. In fact, previous studies approached antioxidant activity in foods with *C*. *vulgaris*, correlating the antioxidant potential with the total amount of incorporation. Increasing the biomass in food composition reveals positive effects in bacterial activity limitation and the promotion of antioxidant activity by a greater presence of phenolics, pigments, and vitamins [78]. This microalgae biomass and/or extracts represent an important alternative with potential application in both the pharmaceutical and food industries [79].

### 3.6. Color Evaluation

One of the main challenges for alternative Clean Label products is to acquire colors that resemble the traditional products. A constant effort to reduce and eliminate the components perceived as unnatural in the consumer’s perspective is part of Clean Label’s agenda. The usage of “artificial colorants” is nowadays associated with less healthy products, demanding the food industry to deeply study colorants of natural origin that can be incorporated into final products [80]. A comparison of Control mayonnaise with artificial colorant (undisclosed ingredient) and CvY mixed system with heterotrophic microalgae is present in Table 6.

Regarding luminosity (L*), there are slight changes, mainly before a pH and salt reduction. This visual aspect can be related to an increase in the droplet size of the emulsions [67]. *C. vulgaris* incorporation also reveals a slight increase in green (a*) and blue (b*) tones. Even though small changes were detected by color tests, in terms of overall visual appearance differences detected by the human eye (∆*E*), only a lowering pH depicts some barely noticeable deviation at close attention. Nevertheless, physical stability has the major impact, and if no instability is noticed, visual appearance slight changes are neglected by the consumers [81].

### 3.7. Sensorial Feedback and Purchase Intent

**Figure 4:**
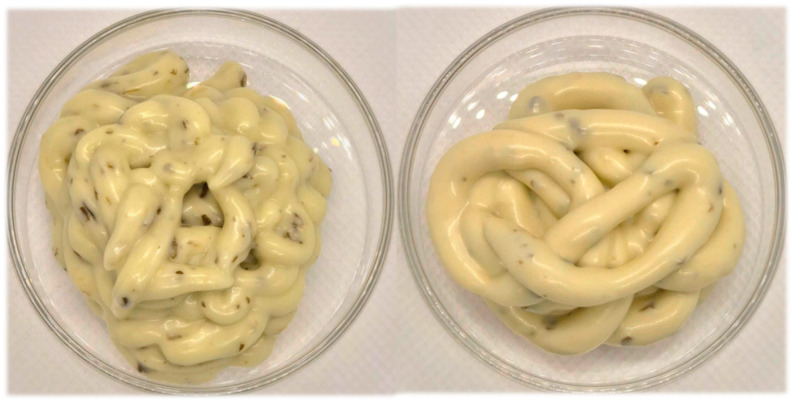
Target coriander mayonnaise (Control) is on the left, and YPE + Microalgae mixed system (CvY) analogue product is on the right.

Consumer reception is subjective and influenced by external factors. Even so, the majority of consumers consider some important variables, such as the yield value, appearance, elasticity, consistency, texture, and flavor, when experiencing mayonnaise. The tasting panel compared the mayonnaises in Figure 4.

**Figure 5:**
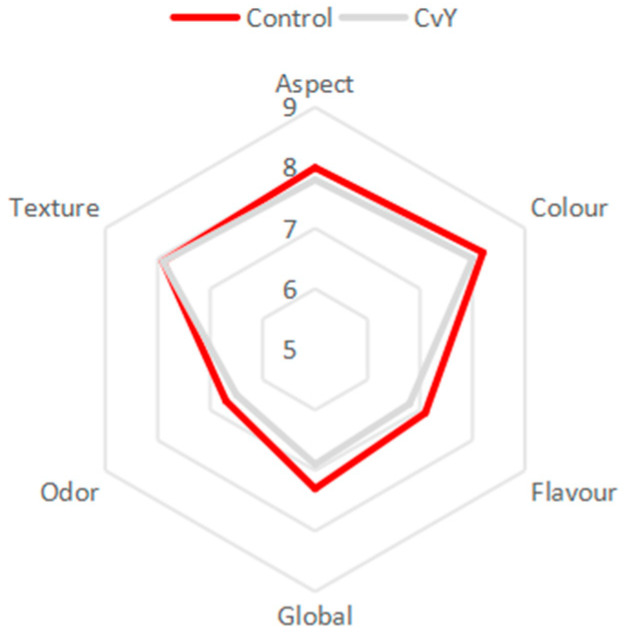
Sensorial analysis preliminary assessment comparison between mayonnaise with egg (Control) and mayonnaise with a mixed system of YPE + Microalgae (CvY).

At a second attempt, factors as viscosity perceived in the mouth infused with intensities of flavor notes sensory profile also reveal to contain a major impact [82]. In Figure 5, it is possible to understand how the tasting panel related Control and CvY in terms of organoleptic characteristics.

**Figure 6:**
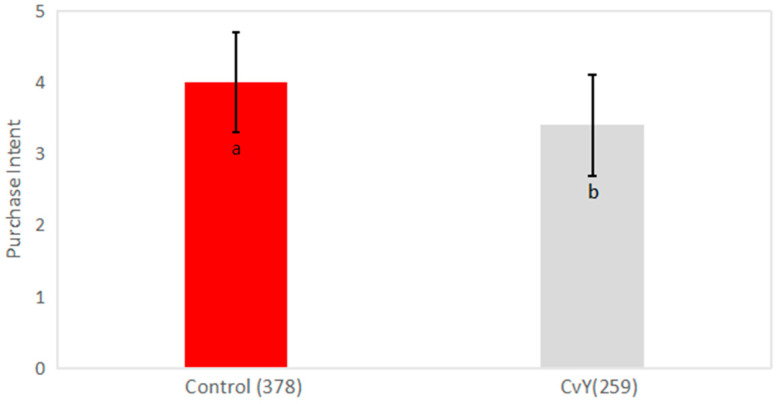
Purchase intent from the tasting panel after a blind sensorial analysis to compare mayonnaise with egg (Control) and mayonnaise with a mixed system of YPE + Microalgae (CvY). Different letters indicate significantly different results (*p* < 0.05) for each column according to Fisher’s test.

Most of the organoleptic profile unveils a balance and similar taste. Focusing on the aspect and color, the application of a heterotrophic microalgae better convinced the consumer since the lack of a green color avoided the initial shock and uncertainty [83]. In terms of flavor, global appreciation, and odor, the microalgae’s distinct character was perceived and ranked below the egg yolk mayonnaise, which evidences that sensory acceptability manifests itself along with the amount of microalgae biomass total incorporation [58]. Ongoing trials of mixed incorporation percentages must be taken into account to balance the purchase intent present in Figure 6, based on the sensory experience.

Although upcoming generations of consumers tend to support plant-based foods in general, this positivism is still far away from a regular purchase intent. Despite the origin of the ingredients, most consumers expect foods to remain similar to their traditional counterparts. Both consumers and the food industry play a role in this matter since alternative products are usually linked to traditional products’ similar responses. If alternative foods fail to reach previously emphasized expectations and turn out to offer an inferior experience, their sales performance will also be inferior [84].

## 4. Conclusions

Mixed systems composed of proteins with synergy to perform emulsifying roles, like yeast protein extract and white *Chlorella vulgaris*, can be used for the stabilization of commercial vegan and clean label mayonnaise, contributing to a new market offering.

Strategies to elaborate a balance consortium between alternative products to successfully mimic and achieve the consumers undistinguished appraisal around these alternative products become even closer to take a place in markets shelfs. Also, focusing on less disruptive organoleptic changes, as offering a similar color by opting for a heterotrophic microalga, seems to nourish a positive acceptance.

The studied systems show a certain robustness against variations in pH and salt concentration, which represents a positive effect to possible unexpected deviations in citric acid or salt amount during the production chain. However, excessively low pH values (around 3.6) should be avoided. In fact, the pH values of commercial emulsions are typically slightly higher. It was also found that increasing the salt concentration positively contributes to the increased degree of structuring of the emulsion, but the limits on salt incorporation in food are becoming more restricted, considering that the maximum recommended daily intake of NaCl is 5 g, according to the guidelines of the World Health Organization (WHO).

Further research is essential to evaluate the digestibility rate and underline the apparent health benefits of the consumption of mayonnaise produced with the mixed system (CvY). Moreover, stability and shelf life must also be taken into consideration. From this initial perspective, CvY systems depict a promising physical similarity, a boost in functional properties, and a good initial response by the tasting panel.

## Figures and Tables

**Table 1:** Coriander mayonnaise formulas (control; 0.8% YPE + 1% *C. vulgaris* (CvY) with different concentrations of citric acid (CvY_ca) and added salt—NaCl (CvY_salt)).

Ingredients	Formulation—g/100 g of Sample					
	Control	CvY	CvY_ca0.1	CvY_ca0.4	CvY_salt0.1	CvY_salt0.5
Colza oil	55.76	55.76	55.76	55.76	55.76	55.76
Distilled water	31.42	31.42	**31.54**	**31.24**	**31.62**	**31.22**
Alcohol vinegar	4	4	4	4	4	4
Modified starch	0.3	0.3	0.3	0.3	0.3	0.3
Egg yolk powder	**1.8**	-	-	-	-	-
YPE	-	**0.8**	0.8	0.8	0.8	0.8
Salt (NaCl)	0.3	0.3	0.3	0.3	**0.1**	**0.5**
Granulated sugar	3.5	3.5	3.5	3.5	3.5	3.5
Coriander	2	2	2	2	2	2
Shade yellow	0.3	0.3	0.3	0.3	0.3	0.3
Potassium sorbate	0.26	0.26	0.26	0.26	0.26	0.26
Citric acid	0.22	0.22	**0.1**	**0.4**	0.22	0.22
Xanthan gum	0.125	0.125	0.125	0.125	0.125	0.125
EDTA	**0.0072**	-	-	-	-	-
White *C. vulgaris*	-	**1.0072**	1.0072	1.0072	1.0072	1.0072
Total (g)	100	100	100	100	100	100

**Table 2:** pH measurement and comparison between Control and mixed system (CvY) samples.

Sample	pH
Control	3.92 ± 0.01 ^a^
CvY	3.89 ± 0.02 ^a^
CvY_ca0.1	4.10 ± 0.03 ^b^
CvY_ca0.4	3.65 ± 0.02 ^c^
CvY_salt0.1	3.91 ± 0.03 ^a^
CvY_salt0.5	3.87 ± 0.02 ^a^

Different letters indicate significantly different results (*p* < 0.05) according to Tukey’s test.

**Table 3:** Williamson model fitting curves parameters comparison between Control and mixed system (CvY) samples.

Samples	η_0_ (Pa.s)	*k* (s)	m	R^2^
Control	9.20 × 10^3^ ± 6.23 × 10^2^ ab	7.57 × 10^1^ ± 1.65 × 10^1^ A	0.89 ± 0.01 ^i^	0.980
CvY	8.96 × 10^3^ ± 1.23 × 10^3^ a	5.57 × 10^1^ ± 1.55 × 10^1^ A	0.92 ± 0.03 ^iI^	0.991
CvY_ca0.1	1.59 × 10^4^ ± 5.96 × 10^3^ b	7.23 × 10^1^ ± 3.74 × 10^1^ A	0.91 ± 0.06 ^iI^	0.989
CvY_ca0.4	7.57 × 10^3^ ± 3.01 × 10^2^ a	4.80 × 10^1^ ± 4.76 × 10^0^ A	0.96 ± 0.02 ^I^	0.989
CvY_salt0.1	6.94 × 10^3^ ± 5.71 × 10^2^ a	4.25 × 10^1^ ± 1.39 × 10^1^ A	0.94 ± 0.01 ^iI^	0.992
CvY_salt0.5	1.14 × 10^4^ ± 2.35 × 10^2^ ab	4.74 × 10^1^ ± 2.23 × 10^0^ A	0.94 ± 0.01 ^iI^	0.996

Different letters indicate significantly different results (*p* < 0.05) for each column according to Tukey’s test.

**Table 4:** TPA parameters comparison between samples.

Samples	Firmness (N)	Elasticity	Cohesiveness	Adhesiveness (N.s)
Control	0.059 ± 0.001 ^a^	0.969 ± 0.001 ^AC^	0.857 ± 0.004 ^i^	0.372 ± 0.005 ^I^
CvY	0.047 ± 0.001 ^b^	0.965 ± 0.006 ^AB^	0.781 ± 0.006 ^ii, iii^	0.256 ± 0.002 ^II^
CvY_ca0.1	0.078 ± 0.002 ^c^	0.956 ± 0.001 ^B^	0.838 ± 0.013 ^i^	0.595 ± 0.007 ^III^
CvY_ca0.4	0.043 ± 0.001 ^d^	0.977 ± 0.003 ^C^	0.783 ± 0.014 ^ii^	0.184 ± 0.003 ^IIII^
CvY_salt0.1	0.039 ± 0.001 ^e^	0.968 ± 0.007 ^ACD^	0.748 ± 0.013 ^iii^	0.181 ± 0.011 ^IIII^
CvY_salt0.5	0.057 ± 0.001 ^d^	0.959 ± 0.005 ^BD^	0.784 ± 0.027 ^ii^	0.361 ± 0.011 ^I^

Different letters indicate significantly different results (*p* < 0.05) for each column according to Tukey’s test.

**Table 5:** Total phenolic compounds (TPC), Radical Scavenging Activity (DPPH), and Ferric Reducing Antioxidant Power (FRAP) comparison between mayonnaise with egg plus EDTA (Control) and mayonnaise with mixed system of YPE plus microalgae (CvY).

Samples	TPC ugG Aeq/g	DPPH ugTEAC/g	FRAP ugTEAC/g
Control	84.21 ± 10.56 ^a^	85.97 ± 12.18 ^A^	50.26 ± 5.31 ^i^
CvY	203.05 ± 2.25 ^b^	117.26 ± 7.96 ^B^	85.47 ± 0.93 ^ii^

Different letters indicate significantly different results (*p* < 0.05) for each column according to Fisher’s test.

**Table 6:** CieLab color space coordinates (L*, a*, and b*) from each sample, plus color changes perception, and final pH.

Samples	L*	a*	b*	∆*E*
Control	84.54 ± 1.42 ^a^	−5.24 ± 0.09 ^A^	24.93 ± 0.46 ^i^	-
CvY	83.62 ± 0.72 ^a^	−5.70 ± 1.42 ^B^	24.19 ± 0.46 ^ii^	1.26
CvY_ca0.1	83.18 ± 0.59 ^ac^	−5.62 ± 0.04 ^B^	24.04 ± 0.36 ^ii^	0.48
CvY_ca0.4	81.36 ± 0.33 ^b^	−5.99 ± 0.04 ^C^	28.42 ± 0.43 ^iii^	4.8
CvY_salt0.1	81.53 ± 1.17 ^b^	−6.13 ± 0.09 ^D^	25.97 ± 0.41 ^iiii^	2.78
CvY_salt0.5	82.22 ± 0.28 ^bc^	−6.14 ± 0.04 ^D^	26.56 ± 0.31 ^iiii^	2.789

Different letters indicate significantly different results (*p* < 0.05) for each column according to Tukey’s test.

## Data Availability

The original contributions presented in this study are included in the article. Further inquiries can be directed to the corresponding author.
